# Botulinum Toxin-A Therapy in Pediatric Urology: Indications for the Neurogenic and Non-Neurogenic Neurogenic Bladder

**DOI:** 10.1100/tsw.2009.146

**Published:** 2009-11-18

**Authors:** Lori Dyer, Israel Franco

**Affiliations:** Department of Pediatric Urology, New York Medical College, Valhalla, USA

**Keywords:** Botulinum Toxin-A, pediatric urology, neurogenic bladder, non-neurogenic neurogenic bladder, external sphincter dyssynergia, overactive bladder, voiding dysfunction

## Abstract

Although, the role of Botulinum Toxin-A in the treatment of the neurogenic and non-neurogenic neurogenic bladder is becoming more defined, this is the first review article to characterize the emerging role of Botulinum Toxin-A in the pediatric urologic population. Injection of Botulinum Toxin-A at the level of the bladder works by inhibiting uninhibited bladder contractions and, possibly, by blocking some of the sensory nerve fibers. In children with sphincter dyssynergy, injection at the level of the urethral sphincter works by inhibiting the involuntary guarding reflex and blocking dyssynergic voiding.

Botulinum Toxin-A was first discovered in the late 1700s and was then used for medicinal purposes in 1897 by Van Ermegam[1]. It is a facultative anaerobe that works by irreversibly binding with the receptors at the presynaptic cholinergic junction and inhibiting the release of acetylcholine at the neuromuscular junction. It acts on the SNAP 25 protein[2]. The decreased levels of acetylcholine inhibit muscle contractility and effectively paralyze muscles. The effect is usually sustained for between 1 and 3 months, until new terminal axons begin to sprout. There are now seven serotypes of Botulinum Toxin-A (A, B, C1, D, E, F, G)[2], which is a derivative of the toxin *Clostridium botulinum*. Botulinum Toxin-A-A is the most widely used for human medicinal purposes. The three commercially available toxins are Botox®, Dysport®, and Myobloc®. Botox and Dysport are A toxins and Myobloc is a B toxin[2]. First approved for use in strabismus patients, FDA-approved indications are still relatively limited and include blepharospasm, cervical dystonia, hyperhydrosis, and temporary improvement of facial lines. There are no approved uses in the pediatric population. Despite its limited FDA approval, many specialties have embraced the toxin to counter various maladies. Off-label use has been popularized for migraine headaches, myofascial pain, dystonia, hemifacial spasm, cerebral palsy, gastrointestinal disorders, pain management, control of autonomic dysreflexia, and chronic constipation (internal anal injection to mirror a myomectomy)[3,4].

The use of Botulinum Toxin-A in human pathology has focused on end-organ therapies to reduce its potentially morbid side effects. Urologists, like most specialists, have recently broadened their interests in Botulinum Toxin-A and have expanded their usage of the toxin, mostly in the subspecialty of neurourology. Doctors are now using it not only for overactive bladder (OAB) and sphincter dyssynergy, but also to lessen chronic increased tonicity of the pelvic floor (and its associated musculature) and for treatment of bladder pain secondary to interstitial cystitis. It seems to have an analgesic effect on chronic inflammation through its effect on c-fibers, substance P, and glutamate[5]. From a urologic perspective, OAB, interstitial cystitis, incontinence, neurogenic detrusor dysfunction, non-neuogenic and neurogenic sphincter dyssynergy, and prostatitis are the present off-label indications.

The pediatric literature is focused on the use of Botulinum Toxin-A in non-neurogenic and neurogenic bladders. The substance is used to treat high bladder pressures and incontinence due to both uninhibited contractions and sphincter dysfunction. As the safety and efficacy of Botulinum Toxin-A continues to be proven, pediatric urologists are beginning to embrace Botulinum Toxin-A as a treatment in patients with refractory voiding dysfunction/daytime incontinence, and non-neurogenic internal and external sphincter dyssynergy.

Doses ranging from 50 to 300 units of Botulinum Toxin-A-A, per dose, have been documented in the pediatric urologic literature, and have been injected into both the detrusor and the urethral sphincter. Variables involved in urologic delivery of the toxin include type, amount, dilution volume (no consensus in the literature), location of the injection, number of injections, depth of the injections, instruments, and type of anesthesia.

It is likely that initial low doses, particularly in children, were arbitrarily used to avoid toxicity. In the adult urologic literature, there are protocols that include injections of up to 1000 SU units of type A botulinum (Dysport)[6] and up to 300 units of Botulinum Toxin-A[5]. Overall, it appears that higher doses are more efficacious. The lethal human dose of *C. botulinum* is approximately 3000 units or 39 units/kg[7]. In order to achieve lethality, the dose must be injected intravascularly. Therapeutic Botulinum Toxin-A should never be injected intravascularly. The side effects documented in the urologic literature include isolated muscle weakness and urinary retention, which uniformly resolve with axonal regeneration of the associated nerves (usually within 1–3 months). In particular, these effects have been noted in patients treated for OAB and are thought to be due to migration of the Botulinum Toxin-A away from the detrusor muscle.

The serious side effects that have been reported in the pediatric population occurred after doses between 6.25 and 32 units/kg of Botulinum Toxin-A (Botox and Dysport), and between 388 and 625 units/kg of Botulinum Toxin type B (Myobloc). None of these problems occurred in children treated with Botulinum Toxin-A for urologic indications. The side effects referenced include iatrogenic botulism[8,9], localized muscle weakness, as well as aspiration pneumonia and respiratory failure requiring intubation and feeding tube placement. However, the 16+ deaths in the pediatric literature were all in children with neurologic disorders, most commonly in children with cerebral palsy. It is not clearly documented whether the aspiration and illnesses were secondary to the Botulinum Toxin-A or the primary pathology (all of which were in children treated for limb muscle spasm). In the pediatric urologic literature, the most serious side effects reported included one girl who had a 10-day episode of urinary retention[10], another girl who had a 2-day episode of urinary retention[11], and one boy who suffered from 2 weeks of flank pain[9]. The situations resolved without any intervention. Other than the data described by Franco et al.[12], who injected the Botulinum Toxin-A transurethrally to the external sphincter, the pediatric literature describes sphincteric Botulinum Toxin-A therapy injection in a transperineal fashion[10]. It can be inferred from the pediatric data extrapolated from transperineal injections for other means that there may be a higher incidence of abscess formation with transperineal vs. transurethral injection.

Botulinum Toxin-A was initially used to treat neurogenic and then non-neurogenic bladders in the adult population. Subsequently, it was introduced into the pediatric population for treatment of the neurogenic bladder[13]. It was first injected at the level of the bladder[14] and then in the urethral sphincter. Finally, the indications have been broadened to include children with refractory voiding dysfunction who have have been injected at the level of the bladder and the urethra. All studies in adults and children have been open label. In patients with uninhibited detrusor contractions, multiple injections are distributed throughout the bladder, totaling between 100 and 300 units. In some studies, adults have been injected in the office-type setting. The typical onset of action can take between 7 and 30 days[5]. In our own patients, the typical onset of action is between 5 and 7 days. In children, the toxin is injected under general anesthesia. The only documented data on duration of action in the pediatric population were described by Petronijevic et al. in 2007[15]. They reported that the average duration of action when Botulinum Toxin-A was injected into the external sphincter was 12–18 weeks. When injected directly into the detrusor, effects can last up to 6 months.

Riccabona and associates[14] injected 15 children with 10 units/kg up to a maximum of 360 units for neurogenic bladder. They all had significant improvement in bladder pressures to a mean duration of 10.5 months. Kajbafzadeh et al.[16] reported on the intravesical injection of Botulinum Toxin-A for neurogenic bowel and bladder in children with myelomeningocele; 73% of all children injected were dry between clean intermittent catheterizations 4 months after the procedure, 88% showed improvement in symptoms overall, and 73% had a decrease in the grade of their reflux. In children with OAB, it is assumed that it is not only the motor effect of the Botulinum Toxin-A of the detrusor, but its effect on the sensory aspect of the reflex arc that is thought to account for the improvement of supersensitivity in OAB and dysfunctional voiding. Hoebeke et al.[9] prospectively reported on 21 patients (10 male, 11 female) who underwent cystoscopic detrusor injection with 100 units of Botulinum Toxin-A for therapy-resistant overactive detrusor. Of the 15 patients who were followed for greater than 6 months, nine children had a complete response to one injection and three children had a partial response. Of the three partial responders, one child had a complete response to a second injection.

Following the success of detrusor injections, the toxin was subsequently used at the level of the external sphincter for the treatment of spinal cord injury patients with detrusor-sphincter-dyssynergy, and also for spinal dysraphism patients suffering from the same fate. Subsequently, Smith et al. and Schulte-Baukloh et al. injected the sphincter to treat adults with OAB[1,17].

Steinhardt et al. pioneered the usage of Botulinum Toxin-A in neurogenically normal children in 1997, when they reported injecting a 7-year-old female with recurrent urinary tract infections (UTIs) who was unresponsive to conventional therapies. The child had dramatic urethral dilation (documented on voiding cystourethrography [VCUG]) secondary to dysfunctional voiding. They injected 20 units total in four locations at the external sphincter. After 18 months, the child was infection free off antibiotics[7].

In an AAP abstract from 1997, Maizels et al.[18] presented a series of 20 females with "lazy bladder" and external sphincter spasticity who were treated with Botulinum Toxin-A injection to the external sphincter on a prospective basis. At 2-year follow-up, the patients had no recurrent UTIs compared with a preprocedural average of three infections per year. However, voiding diaries revealed no statistical differences in voided volumes or voiding intervals. Maizels favored Botulinum Toxin-A injection with 50 units and concomitant urethral dilation. His results portrayed "subjective" improvements in daytime/nighttime urine flow and he postulated that intravesical pressures post-Botulinum Toxin-A were likely decreased. Although the number of UTIs was reduced, Maizels did not achieve total continence in the patients in his study. Radojicic et al.[11] treated eight boys and 12 girls with recurrent UTIs, high postvoid residuals (PVR), and fractional voiding with Botulinum Toxin-A-A, 50–100 units. They used an endoscopic-assisted transperineal approach. Follow-up was between 9 and 18 months, and all children were free of UTIs. One girl had transient (48 h) urinary retention.

Mokhless et al.[19] prospectively evaluated 10 children with non-neurogenic neurogenic bladder. He injected them endoscopically at 3:00, 6:00, and 9:00. All patients except for one were able to void without catheterization immediately after the procedure. Postoperative PVR decreased by 89%, and detrusor leak point pressures and uroflowometry were also considerably improved.

Petronijevic et al.[15] published data describing nine children with PVR >20 ml, prospectively, using a combination of Botulinum Toxin-A to the external sphincter followed by lower urinary tract rehabilitation. Uroflows were performed before and 6 months after treatment. PVR decreased significantly at 6 months, but uroflow parameters were essentially unchanged.

Franco et al.[12] published on Botulinum Toxin-A injection to the external sphincter in children with refractory voiding dysfunction. Prior to Botulinum Toxin-A injection, all patients had failed medical and behavioral therapies. All patients had sphincter dyssynergia documented by preoperative uroflow/electromyography. Average preoperative PVR was 95 cc; 10 of 12 children were dry at the first postoperative visit. Of the remaining two, both had decreased enuresis, and one of the two was dry at the second postoperative visit. Average initial postoperative PVR was 43 cc (range: 0–141 cc) with average second visit PVR equaling 1.8 cc (range: 0–80 cc). Franco's group postulated that the effect of Botulinum Toxin-A is probably a dose-dependent phenomenon, which would explain the discrepancy in results between the two cohorts in Maizel's and Franco's data. In the pediatric literature, prior to 2006, doses ranging between 50 and 100 units were routinely used. Most recently, the literature has shown that 300-unit dosing has increased efficacy with no increase in morbidity[12].

It has been shown by Franco et al.[12] that Botulinum Toxin-A injection to the external sphincter frequently works on children who continue to leak despite complying with a bowel regimen and successfully relaxing their sphincter during biofeedback. This is because they resume the squatting and hoarding that facilitated their initial involuntary guarding reflex when they return home after biofeedback. The effective mechanisms are probably more complex and multifactorial, but simply put, Botulinum Toxin-A produces a reversible chemical sphincterotomy through a minimally invasive procedure. The child is unable to resume dyssynergic voiding because their external sphincter has been defunctionalized. This proposed mechanism of action is further substantiated by the work of Petronijevic et al.[15] and Franco et al.[12] who continued postinjection therapy with lower urinary tract rehabilitation. The inferior results of Mokhless et al.[19] were likely secondary to a lack of patient re-education after the injection therapy .

Botulinum Toxin-A causes forced relaxation of the sphincter and subsequent inhibition of the guarding reflex. It is thought that detrusor hyper-reflexia without detrusor hypertrophy is due to a delay in cortical inhibition (decreased function) of uninhibited bladder contractions via the reticulospinal pathways or the inhibitory feedback from the cerebral cortex (prefrontal cortex or anterior and posterior cingulate gyrus). In the face of uninhibited bladder contractions, the guarding reflex becomes exaggerated, resulting in bladder-sphincter discoordination during voiding. This leads to detrusor thickening and increased bladder instability, and more frequent bladder contractions. This vicious cycle of increased frequency of contractions leads to pelvic floor tightening and classic holding maneuvers (i.e., Vincent's curtsy) that relieve the bladder contractions for a short period of time. Injecting Botulinum Toxin-A to the urethral sphincter causes sensory interruption of the feedback loops/reflex arc. Thus, in children whose continence during uninhibited contractions is maintained by external sphincter contraction, there may be initial incontinent episodes after Botulinum Toxin-A injection until they relearn normal voiding patterns[10].

Most of the patients described by Franco et al.[12] documented external sphincter dyssynergy on UDS/EMG studies; however, several of the patients actually had internal sphincter dyssynergy. It would seem counterintuitive that the patients with internal sphincter dyssynergy (smooth muscle) would respond to Botulinum Toxin-A, when the toxin historically has been described as working at the presynaptic neuromuscular junction to inhibit solely striated muscle. However, research done on the rat bladder by Smith et al.[1] showed that Botulinum Toxin-A decreases levels of norepinepherine in addition to decreasing levels of acetylcholine. It is this decrease in norepinepherine that may have clinical benefit in inhibiting smooth muscle dyssynergy by decreasing sympathetic tone in the internal sphincter in children. This same mechanism is what assists with limiting afferent nerve excitability.

In the adult literature, there have been case reports of Botulinum Toxin-A failure secondary to the possibility of antibody formation after the primary injection. This was determined after the measurement of antibody levels[20]. Antibody levels have not been sampled in the pediatric population, but the current thinking regarding treatment failure is based on the concept that the failure is related to a complex cortical level problem and not a simple serologic problem. In the data from pediatric literature, one of the only recurrent failures was a subject with complex neuropsychiatric problems[12]. His failure was likely due to the complexity of his primary problem.

Using Botulinum Toxin-A at the level of the sphincter treats a symptom of a sometimes multifactorial problem: external sphincter dyssynergia. The problem is generally treated on a continuum and refractory voiding dysfunction is frequently associated with neuropsychiatric problems. Botulinum Toxin-A is the last urologic option. When a patient is even refractory to this treatment, it is likely that the child's problems are inherently more complex. These children with neuropsychiatric problems need to be approached concomitantly from a central perspective. Botulinum Toxin-A, of its own volition, may treat the symptoms and even lead to retraining of the voiding reflexes of many children with dysfunctional voiding. However, in children who are refractory to Botulinum Toxin-A therapy, the primary neuropsychiatric disorder needs to be treated. The child who failed three Botulinum Toxin-A injections had symptoms of recurrent epididymitis and suffered from ADHD. He was eventually treated with Adderal and his symptoms improved.

On the horizon, it would be interesting to obtain more studies to evaluate children treated with Botulinum Toxin-A (neurogenic and non-neurogenic would be separated) in a randomized, prospective clinical trial. Although there are several series that attempted this, the series numbers have been very small[14,15]. Ideally, it would be useful to perform videourodynamics on all of these patients at uniform intervals after injection. Unfortunately, our current thinking is limited to results composed of subjective data and post-therapeutic PVR measurements. More prospective trials would give us the opportunity to standardize our results. Utilizing some of the validated continence satisfaction questionnaires that have been instrumented in the adult urologic Botulinum Toxin-A population[17] could be useful. Unfortunately, there are very few parents of children with non-neurogenic voiding dysfunction who will agree to have routine invasive testing, such as videourodynamics, performed after their child has shown improvement.

In conclusion, Botulinum Toxin-A injection of the detrusor and the external sphincter appears to be a safe and efficacious way to treat refractory neurogenic and non-neurogenic voiding dysfunction in children. Other treatment options that have been previously used in the adult population, including combined sphincteric and trigonal injection, are currently being tried in the pediatric population. Long-term follow-up of all options is necessary and repeat injections may be required in selected patients.
